# Attitudes of People in the UK with HIV Who Are Antiretroviral (ART) Naïve to Starting ART at High CD4 Counts for Potential Health Benefit or to Prevent HIV Transmission

**DOI:** 10.1371/journal.pone.0097340

**Published:** 2014-05-28

**Authors:** Alison J. Rodger, Andrew Phillips, Andrew Speakman, Richard Gilson, Martin Fisher, Ed Wilkins, Jane Anderson, Margaret Johnson, Rebecca O'Connell, Simon Collins, Jonathan Elford, Lorraine Sherr, Fiona C. Lampe, for the ASTRA (Antiretrovirals, Sexual Transmission Risk and Attitudes) Study Group

**Affiliations:** 1 University College London, London, United Kingdom; 2 Brighton and Sussex University Hospitals NHS Trust, Brighton, United Kingdom; 3 Pennine Acute Hospitals NHS Trust, Manchester, United Kingdom; 4 Homerton University Hospital NHS Foundation Trust, London, United Kingdom; 5 Royal Free NHS London Foundation Trust, London, United Kingdom; 6 Newham University NHS Hospital Trust, London, United Kingdom; 7 HIV i-Base, London, United Kingdom; 8 City University London, London, United Kingdom; University of Pennsylvania School of Medicine, United States of America

## Abstract

**Objective:**

To assess if a strategy of early ART to prevent HIV transmission is acceptable to ART naïve people with HIV with high CD4 counts.

**Design:**

ASTRA is a UK multicentre, cross sectional study of 3258 HIV outpatients in 2011/12. A self-completed questionnaire collected sociodemographic, behavioral and health data, and attitudes to ART; CD4 count was recorded from clinical records.

**Methods:**

ART naïve participants with CD4 ≥350 cells/µL (n = 281) were asked to agree/disagree/undecided with the statements (i) I would want to start treatment now if this would slightly reduce my risk of getting a serious illness, and (ii) I would want to start treatment now if this would make me less infectious to a sexual partner, even if there was no benefit to my own health.

**Results:**

Participants were 85% MSM, 76% white, 11% women. Of 281 participants, 49.5% and 45.2% agreed they would start ART for reasons (i) and (ii) respectively; 62.6% agreed with either (i) or (ii); 12.5% agreed with neither; 24.9% were uncertain. Factors independently associated (p<0.1) with agreement to (i) were: lower CD4, more recent HIV diagnosis, physical symptoms, not being depressed, greater financial hardship, and with agreement to (ii) were: being heterosexual, more recent HIV diagnosis, being sexually active.

**Conclusions:**

A strategy of starting ART at high CD4 counts is likely to be acceptable to the majority of HIV-diagnosed individuals. Almost half with CD4 >350 would start ART to reduce infectiousness, even if treatment did not benefit their own health. However a significant minority would not like to start ART either for modest health benefit or to reduce infectivity. Any change in approach to ART initiation must take account of individual preferences. Transmission models of potential benefit of early ART should consider that ART uptake may be lower than that seen with low CD4 counts.

## Introduction

Untreated HIV-infection causes progressive CD4 depletion and increasing risk of AIDS-defining illnesses and death. It is uncontroversial that antiretroviral treatment (ART) should be started before immunosuppression is advanced and the risk of serious illness becomes significant. However there is no clear consensus regarding precisely when (at what CD4 count) an HIV-infected person should start ART. In the current (2012) European AIDs Clinical Society guidelines, commencement of ART is recommended with a CD4 count below 350 cells/µL [1]. With asymptomatic HIV infection and a CD4 count between 350–500 cells/µL it is recommended that ART be considered, but deferred at counts >500 cells/µL [1]. Similar recommendations on CD4 thresholds are made by the UK 2012 guidelines [2]. In July 2012, WHO guidelines, which are influential for treatment programmes in developing countries, recommended starting ART at a threshold of 500 CD4 cells/µL [3]. In contrast, in 2012 the U.S. Guideline Committee, the DHHS [4], and the IAS-USA [5], recommended that ART be initiated in all people with HIV, regardless of CD4 count. Recommendations to start at CD4 counts above 350 cells/µL are based on evidence from observational studies and trials with deferral strategies to start below 250 cells/µL, and remain controversial [6].

Randomized Control Trial (RCT) evidence of the clinical effectiveness of starting ART (at higher CD4 counts) is currently being evaluated in the START trial [7], an international trial that has almost fully recruited 4600 participants and also in the TEMPRANO trial [8] - now fully recruited with 1600 participants in Côte D'Ivoire. In START, HIV-infected individuals with CD4 count >500 cells/µL are randomized to immediate ART initiation or deferral until CD4 count has declined to 350 cells/µL. The results of TEMPRANO are expected in 2015 and START in 2016. However, even if clinical benefit were shown in randomized evaluation, the absolute risk reduction of ill health associated with early ART is likely to be modest, given the low risk of serious illness with CD4 counts >350 cells/µL [9].

However, the potential individual clinical benefit of earlier treatment is not the only factor under consideration in the debate regarding when people diagnosed with HIV should start ART. There is increasing interest in a policy of early ART to reduce HIV transmission. Observational studies report that people on ART with a suppressed viral load have markedly reduced infectiousness [10]–[12]. The HPTN 052 RCT prospectively evaluated the effect of ART on prevention of HIV transmission to HIV negative heterosexual partners. This study reported 96% reduction of transmissions in serodifferent couples (CD4 count 350–550 cells/µL at entry) assigned to the early ART arm [13] compared to those where ART was differed to CD4 count <250 cells/µL. The results of this landmark trial in 2011 dramatically increased interest in the use of ART for prevention. UK, European, WHO and American guidelines now recommend explaining to all HIV positive individuals that there is a beneficial effect of ART on infectiousness and that ART be offered to anyone who wants to take it to reduce transmission risk [2], [4], [14], [15].

The question arises as to whether a policy of ART for all people diagnosed with HIV should be adopted, even in the absence of evidence of significant individual health benefit.

There remain unanswered questions prior to widespread roll out of ART for prevention of transmission for people with higher CD4 counts. Issues include the durability of the prevention effect and, especially in a non-trial setting, the lack of precise transmission risk estimates for sex without the use of condoms (particularly for anal sex), long term adherence and tolerability of treatment, and the potential for transmission of resistant strains. In addition there are issues in resource-restrained environments with insufficient roll out of ART to those under 350 cells/µL [16]. One of the most significant unknowns, however, is the extent to which a policy of early ART would be acceptable to people with HIV, particularly if there was little or no individual health benefit to starting therapy at higher CD4 counts.

The aim of this study was to use data from the ASTRA (Antiretroviral, Sexual Transmission Risk and Attitudes) UK multi-centre questionnaire study of outpatients with HIV, to investigate attitudes to earlier ART (i) for modest potential health benefit, and (ii) to reduce infectiousness, among ART-naïve HIV diagnosed individuals with CD4 count ≥350cells/µL.

## Methods

### Ethics statement

Ethical approval for the ASTRA study was obtained via the National Research Ethics Service (10/H0720/70).

ASTRA was a cross-sectional self-administered questionnaire study in 3258 outpatients recruited from February 2011 to December 2012 at eight UK outpatient HIV clinics. The overall response rate was 64%. The questionnaire collected information on demographic, social, lifestyle, HIV and health-related factors, in addition to beliefs about sexual transmission risk, and attitudes to early ART (for participants who were ART naïve only). Most recent CD4 count and viral load were recorded from clinical records for all participants. In addition, 2983 (92%) of the 3258 participants gave consent for linkage of their questionnaire data with routine clinical information from their HIV clinic records. The full methods for the ASTRA study are published elsewhere [17].

Of 3258 participants, 3202 gave information on ART status of which 364 (11.4%) reported that they had never taken ART. Of these 364, a further 6 participants were excluded as linked clinical data recorded ART use at the time of the questionnaire. Of the remaining 358 participants 281 had a CD4 cell count of ≥350cells/µL (according to CD4 count recorded in the study log) and are included in this analysis (8.6% of the total ASTRA sample).

Participants who were ART naïve were asked the extent to which they agreed with the following statements: (i) I would want to start treatment now if this would slightly reduce my risk of getting a serious illness; (ii) I would want to start treatment now if this would make me less infectious to a sexual partner, even if there was no benefit to my own health

Response options were: strongly agree; tend to agree; undecided or no opinion; tend to disagree; strongly disagree. Factors associated with agreement (‘strongly agree’ or ‘tend to agree’, versus all other responses) to statements (i) (starting for modest health benefit) and (ii) (starting to reduce infectiousness) were assessed.

Factors considered were demographic and social factors [age group, gender/sexuality, ethnicity, country of birth, employment status, education, financial hardship, current partner, children, social support (assessed by a modified version of the Duke UNC Functional Social Support Questionnaire [18]); HIV-related factors [time since HIV diagnosis, latest CD4 count]; health-related factors [hepatitis C diagnosis ever, recent diagnosed sexually transmitted infection; depressive symptoms (‘major depressive disorder’ according to PHQ-9 [19]; moderate or high physical symptom distress (assessed by a modified version of the Memorial Symptoms Assessment Scale Short-Form [20]); lifestyle factors [smoking status, alcohol use (assessed by modified WHO AUDIT-C questionnaire) and alcohol dependency (assessed by CAGE questionnaire [21], recent recreational drug use]; recent sexual activity; ART optimism (agreement with the statement ‘better HIV treatment means people are less worried about infecting others’).

Chi-squared tests and chi-squared tests for trend were used to assess the univariable associations of the above factors with agreement to attitude questions (i) and (ii). Logistic regression analysis was used to produce unadjusted odds ratios with 95% confidence intervals, and to assess multivariable associations. The final multivariable models were developed as follows: first all factors with p≤0.1 in univariable associations were included in an intermediate model; next each of the other factors was added to the intermediate model individually, and retained if p≤0.1. Results are presented as adjusted odds ratios (OR) with 95% confidence intervals. Likelihood ratio tests were used to produce p-values. ART optimism variables were not considered as potential factors to be included in the multivariable model as they were considered too similar to the outcomes of interest (attitudes to early ART).

## Results

Overall, 281 ART naïve participants were included in this analysis (Table 1). Of these 85.0% were MSM, 3.9% heterosexual men and 11.0% women. The mean age was 39.5 years (range 20 to 71 years). Ethnicity was 76.2% white, 8.5% Black African and 15.3% other ethnicity. CD4 count was 350–499 mm^3^, 500–699 mm^3^ and ≥700 mm^3^ in 35.2%, 38.1% and 26.7% respectively. Overall 74.4% were in employment and 46.3% university educated. In terms of sufficient money to cover basic needs, less than half (44.8%) reported always having sufficient money to cover basic needs. Half (50.5%) had a stable partner of whom 47.2% were also HIV positive. In terms of health behaviours; 43.4% were current smokers, 24.2% had hazardous or harmful drinking on CAGE screen and 53.0% had used recreational drugs in the previous 3 months.

**Table 1:** Association of factors with (i) wanting to start ART for modest health benefit* and (ii) wanting to start ART to reduce infectiousness* among 281 ART naïve people with CD4≥350/cells/µL.

		N	n (%) who agree with (i) would start for modest health benefit	P value	n (%) who agree with (ii) Would start to reduce infectiousness	P value
TOTAL		281	139 (49.5)		127 (45.2)	
Gender/Sexuality group	MSM	239	113 (47.3)	0.080	103 (43.1)	0.092
	Heterosexual men and women	42	26 (61.9)		24 (57.1)	
Ethnicity	White	214	101 (47.2)	0.17	94 (43.9)	0.44
	Black African/other ethnicity	67	38 (56.7)		33 (49.3)	
Born in UK?	Yes	170	80 (47.1)	0.48	77 (45.3)	0.82
	No	107	55 (51.4)		47 (43.9)	
Age group (Years)	≤30	44	19 (43.2)	0.64	21 (47.7)	0.88
	30–50	196	100 (51.0)		90 (45.9)	
	>50	31	15 (48.4)		13 (41.9)	
Employment	Employed	209	98 (46.9)	0.14	93 (44.5)	0.69
	Other	72	41 (56.9)		34 (47.2)	
Education	Uni education	130	65 (50.0)	0.78	62 (47.7)	0.42
	Other	147	71 (48.3)		63 (42.9)	
‘Money to cover basic needs?’ (financial hardship)	Yes, always	126	50 (39.7)	0.004 trend	53 (42.1)	0.22 trend
	Yes, mostly	82	44 (53.7)		38 (46.3)	
	Yes, sometimes	43	24 (55.8)		18 (41.9)	
	No	27	18 (66.7)		16 (59.2)	
Stable partner	Yes, HIV +ve	67	33 (49.3)	0.19	25 (37.3)	0.30
	Yes, HIV –ve	75	31 (41.3)		37 (49.3)	
	No partner	138	75 (54.3)		65 (47.1)	
Have children	Yes	43	26 (60.5)	0.12	22 (51.2)	0.41
	No	237	113 (47.7)		105 (44.3)	
Social support group (measure of supportive relationships, modified Duke UNC FSSQ)	1: High	79	31 (39.2)	0.017 trend	31 (39.2)	0.76 trend
	2–4	171	89 (52.0)		85 (49.7)	
	5: Low	27	17 (63.0)		9 (33.3)	
Time since HIV diagnosis	≤3 months	30	17 (56.7)	0.019 trend	14 (46.7)	0.001 trend
	3 months–2 years	91	52 (57.1)		53 (58.2)	
	2–5 years	79	36 (45.6)		38 (48.1)	
	>5 years	78	31 (39.7)		21 (26.9)	
CD4 count/cells/µL from clinic records	350–499	99	54 (54.5)	0.090 trend	43 (43.4)	0.46 trend
	500–699	107	54 (50.5)		47 (43.9)	
	≥700	75	31 (41.3)		37 (49.3)	
Smoking status	Current	122	60 (49.2)	0.92	53 (43.4)	0.61
	Ex	68	35 (51.5)		29 (42.6)	
	Never	91	44 (48.4)		45 (49.5)	
Heavy drinking# (modified WHO AUDIT-C)	No	247	124 (50.2)	0.51	108 (43.7)	0.18
	Yes	34	15 (44.1)		19 (55.9)	
Alcohol misuse (by CAGE)	No	213	105 (49.3)	0.92	93 (43.7)	0.36
	Yes	68	34 (50.0)		34 (50.0)	
Recreational drug past 3 months	No	132	62 (47.0)	0.43	56 (42.4)	0.38
	Yes	149	77 (51.7)		71 (47.7)	
Major depression symptoms (by PHQ-9)	No	239	121 (50.6)	0.35	108 (45.2)	0.99
	Yes	42	18 (42.9)		19 (45.2)	
Moderate/high physical symptom distress$ (modified MSAS-SF questionnaire)	No	239	111 (46.4)	0.016	109 (45.6)	0.74
	Yes	42	28 (66.7)		18 (42.9)	
Hepatitis C diagnosis ever	No	254	127 (50.0)	0.58	118 (46.5)	0.19
	Yes	27	12 (44.4)		9 (33.3)	
STI in past 3 months	No	237	115 (48.5)	0.46	106 (44.7)	0.71
	Yes	44	24 (54.5)		21 (47.7)	
Sexually active (vaginal/anal sex) in past 3 months	No	61	28 (45.9)	0.53	21 (34.4)	0.056
	Yes	220	111 (50.5)		106 (48.2)	
Sexual activity category in past 3 months:	No vaginal/anal sex status partner(s)	61	28 (45.9)	0.36	21 (34.4)	0.20
	Condom-protected sex only	86	49 (57.0)		45 (52.3)	
	Condom-less sex HIV+ve partners only	75	33 (44.0)		34 (45.3)	
	Condom-less sex HIV-ve or unknown status partner(s)	51	24 (47.1)		23 (45.1)	
Agree ART mean people less worried infecting others (ART optimism)	Yes	100	61 (61.0)	0.005	60 (60.0)	<0.001
	No/unsure	175	76 (43.4)		64 (35.6)	

* tend to agree or strongly agree combined, versus undecided/tend to disagree/strongly disagree combined.

P values by chi-squared tests, and chi-squared tests for trend.

# usual alcohol intake of at least 5 units 4 or more times a week, or at least 10 units 2–3 times a week.

$ score of 10 or more, based on total distress score for 19 physical symptoms, each scored 0 (symptom not present or no distress) to 4 (distressed very much by symptom).

**Figure 1:**
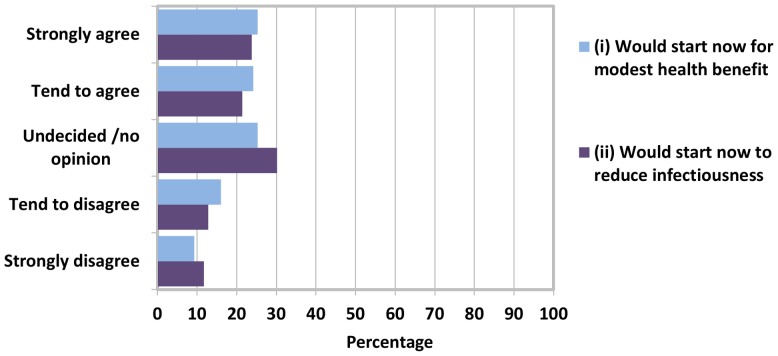
Attitudes to early ART among 281 ART naïve individuals with CD4 ≥350 cells/µL.

Figure 1 shows attitudes to starting early ART. A quarter (25.3%) of participants strongly agreed that they would want to start ART now to slightly reduce risk of serious illness, while a similar number (24.2%) tended to agree with this statement. A further quarter (25.3%) was undecided, and the remaining quarter disagreed (tend to disagree 16%, strongly disagree: 9.3%). A similar pattern of response was apparent for wanting to start ART now to reduce infectiousness even if there was no health benefit, although the proportions who were undecided was somewhat higher (30.2%).

Combining the ‘strongly’ and ‘tend to’ responses, the percentages of participants agreeing that they would want to start ART now to: (i) slightly reduce risk of serious illness was 49.5% (139/281) and (ii) to reduce infectiousness even if there was no health benefit was 45.2% (127/281). Overall, 32.0% (90/281) of participants agreed with both statements (i) and (ii), 30.6% (86/291) agreed with one of the two statements, 12.5% (35/281) disagreed with both statements, and 24.9% (70/281) were uncertain.

Table 1 shows the unadjusted associations of agreement with statements (i) and (ii) with other factors (demographic, socio-economic, health-related, lifestyle, sexual activity and ART optimism). In unadjusted analysis, ‘wanting to start ART for modest health benefit’ was associated with being heterosexual compared to MSM (p = 0.080), greater levels of financial hardship (p = 0.004, test for trend), low levels of social support (p = 0.017, trend), having a lower CD4 count (p = 0.09, trend), shorter length of time since HIV diagnosis (p = 0.019, trend), having moderate or high physical symptom distress scores (p = 0.016) and agreeing that ART meant people were less worried about infecting others (p = 0.005). Univariable associations with starting ART for (ii) to reduce infectiousness even if there was no health benefit were being heterosexual compared to MSM (p = 0.092), shorter length of time since HIV diagnosis (p = 0.001, trend), being sexually active in the past 3 months (p = 0.056) and agreeing that ART meant people were less worried about infecting others (p<0.001). Among participants who were sexually active, there was no evidence that the proportion wanting to start to reduce infectiousness differed between those having condom-protected compared to condom-less sex (Table 1).

Table 2 and 3 show factors included in the final multivariable models for agreement with statements (i) and (ii). The ART optimism variable was not included in these models. Factors that remained independently associated (p<0.1) with wanting to start ART for (i) modest health benefit were: having a lower CD4 count; a more recent HIV diagnosis; financial hardship; presence of moderate or high physical symptom distress. In addition, absence of major depressive disorder by PHQ-9 was significantly associated with wanting to start ART for health benefit once the other factors were included in the model. Factors independently associated (p≤0.1) with (ii) wanting to start ART to reduce infectiousness even if there was no health benefit were: having a more recent HIV diagnosis; being heterosexual compared to MSM; being currently sexually active.

**Table 2:** Independent association of factors with (i) wanting to start ART for modest health benefit* in ART naïve people with CD4≥350/cells/µL.

		N	n (%) who agree*	P value	Unadjusted odds ratio (95% CI)	Adjusted odds ratio (95% CI) # (N = 275)	P value for adjusted model#
TOTAL		281	139 (49.5)				
‘Money to cover basic needs?’(financial hardship)	Yes, always	126	50 (39.7)	0.004 trend	1	1	0.007 trend
	Yes, mostly	82	44 (53.7)		1.8 (1.0, 3.1)	1.8 (1.0, 3.3)	
	Yes, sometimes	43	24 (55.8)		1.9 (1.0, 3.9)	2.2 (1.0, 4.7)	
	No	27	18 (66.7)		3.0 (1.3, 7.3)	2.8 (1.1, 7.5)	
Time since HIV diagnosis	≤3 months	30	17 (56.7)	0.019 trend	1.0 (0.4, 2.3)	0.8 (0.3, 2.0)	0.038 trend
	3 months–2 years	91	52 (57.1)		1	1	
	2–5 years	79	36 (45.6)		0.6 (0.3, 1.2)	0.6 (0.3, 1.1)	
	>5 years	78	31 (39.7)		0.5 (0.3, 0.9)	0.4 (0.2, 0.8)	
CD4 count cells/µL	350–499	99	54 (54.5)	0.090 trend	1	1	0.044 trend
	500–699	107	54 (50.5)		0.8 (0.5, 1.5)	0.8 (0.4, 1.4)	
	≥700	75	31 (41.3)		0.6 (0.3, 1.1)	0.5 (0.3, 1.0)	
Major depression (PHQ-9 MDD)	No	239	121 (50.6)	0.35	1	1	0.019
	Yes	42	18 (42.9)		0.7 (0.4, 1.4)	0.4 (0.2, 0.9)	
Moderate/high physical symptom distress$ (modified MSAS questionnaire)	No	239	111 (46.4)	0.016	1	1	0.046
	Yes	42	28 (66.7)		2.3 (1.2, 4.6)	2.3 (1.0, 5.1)	

*tend to agree or strongly agree combined, versus undecided/tend to disagree/strongly disagree combined.

# using logistic regression analysis; p values by likelihood ratio test.

$ score of 10 or more, based on total distress score for 19 physical symptoms, each scored 0 (symptom not present or no distress) to 4 (distressed very much by symptom).

**Table 3:** Independent association of factors with (ii) wanting to start ART to reduce infectiousness* among 281 ART people who were ART naïve with CD4≥350cells/µL.

		N	n (%) who agree*	P value	Unadjusted odds ratio (95% CI)	Adjusted odds ratio (95% CI): # (N = 278)	P value for adjusted model#
TOTAL		281	127 (45.2)				
Gender/sexuality	MSM	239	103 (43.1)	0.092	1	1	0.058
	Heterosexual men and women	42	24 (57.1)		1.8 (0.9, 3.4)	2.0 (1.0, 4.1)	
Time since HIV diagnosis	≤3 months	30	14 (46.7)	0.001 trend	0.6 (0.2, 1.4)	0.6 (0.2, 1.5)	0.001 trend
	3 months–2 years	91	53 (58.2)		1	1	
	2–5 years	79	38 (48.1)		0.7 (0.4, 1.2)	0.7 (0.3, 1.3)	
	>5 years	78	21 (26.9)		0.3 (0.1, 0.5)	0.2 (0.1, 0.5)	
Sexually active in past 3 months	No	61	21 (34.4)	0.056	1	1	0.025
	Yes	220	106 (48.2)		1.8 (1.0, 3.2)	2.0 (1.1, 3.9)	

* tend to agree or strongly agree combined, versus undecided/tend to disagree/strongly disagree combined;

# using logistic regression analysis; p values by likelihood ratio test.

## Discussion

This is the first study to our knowledge to assess the acceptability of ART at high CD4 counts to reduce the risk of transmission to others in people with HIV in a general clinic setting. We found that almost one half (45%) of HIV-diagnosed individuals not on ART with CD4 ≥350cells/µL reported that they would want to start early ART to reduce infectiousness, even with no health benefit and that half (50%) would want to start for modest health benefit i.e. a small reduction in risk of serious illness. Overall, 62.6% would want to start ART for either or both reasons, 12.5% indicated they would not want to start for either reason, while 24.9% were uncertain. People who had lived longer with HIV were much less likely to want to start ART for either reason, and those with higher CD4 counts were less likely to want to start for modest health benefit.

There is now overwhelming evidence of the reduction in infectiousness through heterosexual sex when the HIV positive partner is on ART with a suppressed VL, although evidence for reduction in infectiousness through anal sex is still lacking [22]. It is therefore entirely appropriate that this should be explained to all people with HIV and ART offered to those who wish to take it to reduce infectiousness; a stance reflected in current guidelines in this area [1], [3], [4], [14]. It is, however, a different matter to advance a general recommendation of ART for all those with diagnosed HIV to prevent transmission as a public health measure. Indeed a key factor in determining in the acceptability and therefore the success of such a strategy of ‘ART for prevention of HIV transmission’ must be the willingness of people who are HIV positive to voluntarily take ART at higher CD4 counts for what may be a minimal absolute reduction in personal risk of adverse clinical outcomes

It is also important to consider the generalizability of ‘when to start’ RCTs which may show modest health gain. The success of ART is highly dependent on readiness to start and motivation to adhere to treatment. Trial participants are a selected group who necessarily must agree in principle to randomisation to early treatment, and may well have a preference for early ART, or high motivation to adhere to ART. The promise of a slight health gain may not universally be viewed by people who are HIV positive as a sufficient incentive to start ART at high CD4 counts and individual preferences and concerns need to be taken into account.

We found that those with high CD4 counts (>700 cells/µL) were much less likely to want to start ART for a small reduction in the risk of serious illness, most likely reflecting their perception of their current low risk of such health problems. We also found a strong association with financial hardship. People experiencing greater levels of financial hardship were considerably more likely to want to start ART for modest health benefit perhaps indicating the vulnerability of their situation and the unwillingness to consider even a small health risk due to the potential impact on an already precarious financial situation. We also found that those who scored higher for physical symptom distress were more likely to wish to start ART for modest health benefit, which may indicate optimism about the potential health benefits of ART, or that they perceive that their current symptoms may be due to uncontrolled HIV infection. Conversely participants who met criteria for major depressive symptoms were less likely to wish to start ART for health benefit, perhaps indicating a reduced interest or concern in their health, or lack of motivation to take regular medication or a negative low mood meant that positive health or future benefits did not resonate as important in their life choices.

People who had lived longer with diagnosed HIV were less likely to want to start immediate ART, either for reasons of health or reduced infectivity. Individuals who have lived longer with untreated HIV and potentially through an era where early treatment was not on the agenda and there was a great awareness of drug toxicities, may perceive their current health status as good without being on ART. The association with time since HIV diagnosis was particularly strong for not wanting to start to reduce infectiousness. Individuals with longstanding infections have had years to assess issues of infectiousness and potentially adapt sexual practices and other risks, so may be less interested in ART to reduce infectiousness. Conversely individuals diagnosed recently have been diagnosed in an era where early ART is very much on the agenda, new treatments are safe and tolerable, and ART may be generally more favourably perceived.

Modelling work to assess the impact of ART on population transmission has considered issues such as adherence to ART and sexual risk behaviour change on ART, but the potential lower uptake of ART has not generally been considered, presumably partly due to lack of data on the acceptability of taking ART to individuals with high CD4 counts [23]. Our results are consistent with the view that uptake and adherence might well be somewhat lower if ART is initiated in people where there is not a clear and immediate personal health risk.

One potential limitation of our study is that the study was conducted only in UK HIV outpatients, and the majority of participants were MSM. Results may not be generalizable to other settings, in particular to resource-limited settings. We had limited power to assess differences according to gender/transmission group. Our data suggested demographic variation in likelihood of wanting to start immediate ART, with some evidence that heterosexual men and women were more likely to want to start than MSM. However confidence intervals for this estimate were wide. In addition, the views we are reporting were expressed in 2011/12 and we should consider that attitudes to early ART to prevent transmission might change rapidly as the publicity around treatment for prevention gains momentum. Likewise, results from ongoing randomized trials could cause a further shift in views. In addition, in this large quantitative study we were not able to explore in depth motivations for responses to the questions regarding early ART. Further qualitative research in this area would be beneficial.

The only other data that we are aware of in this area is from the HPTN 052 trial itself, which was conducted, largely in heterosexual couples in 9 countries [13]. After the HPTN 052 study results of 96% reduction in HIV transmission in the early treatment arm were made public, ART was offered to all HIV positive participants in the delayed arm. However, even with the knowledge that ART had a dramatic effect on risk of transmission, 20% of participants in the deferred ART group - who had agreed at study entry to possible randomisation to initiate ART at higher CD4 count - chose not to initiate ART [24]. This indicates that even in this highly select study population a significant proportion of people feel reluctance to initiate ART primarily to prevent transmission.

In conclusion, a strategy of starting ART at high CD4 counts is likely to be acceptable to the majority of HIV-diagnosed individuals. Nearly two thirds of ART naïve respondents with CD4>350/mm^3^ said they would start immediate ART either for modest health benefit or to reduce infectiousness and almost one half would start to reduce infectiousness even if there were no health benefits. Nonetheless, our results suggest that any change in ART initiation must carefully consider individual needs and preferences. It is unlikely to be universally acceptable to initiate ART at a high CD4 count, even if modest health benefits become established in randomized trials. This information on potential uptake of earlier ART should be incorporated into transmission models of the potential population benefit of early ART.
